# Behavioural Responses of Dusky Dolphin Groups (*Lagenorhynchus obscurus*) to Tour Vessels off Kaikoura, New Zealand

**DOI:** 10.1371/journal.pone.0041969

**Published:** 2012-07-23

**Authors:** David Lundquist, Neil J. Gemmell, Bernd Würsig

**Affiliations:** 1 Department of Anatomy, University of Otago, Dunedin, New Zealand; 2 Department of Marine Biology, Texas A&M University at Galveston, Galveston, Texas, United States of America; Biodiversity Insitute of Ontario – University of Guelph, Canada

## Abstract

**Background:**

Commercial viewing and swimming with dusky dolphins (*Lagenorhynchus obscurus*) near Kaikoura, New Zealand began in the late 1980s and researchers have previously described changes in vocalisation, aerial behaviour, and group spacing in the presence of vessels. This study was conducted to assess the current effects that tourism has on the activity budget of dusky dolphins to provide wildlife managers with information for current decision-making and facilitate development of quantitative criteria for management of this industry in the future.

**Methodology/Principal Findings:**

First-order time discrete Markov chain models were used to assess changes in the behavioural state of dusky dolphin pods targeted by tour vessels. Log-linear analysis was conducted on behavioural state transitions to determine whether the likelihood of dolphins moving from one behavioural state to another changed based on natural and anthropogenic factors. The best-fitting model determined by Akaike Information Criteria values included season, time of day, and vessel presence within 300 m. Interactions with vessels reduced the proportion of time dolphins spent resting in spring and summer and increased time spent milling in all seasons except autumn. Dolphins spent more time socialising in spring and summer, when conception occurs and calves are born, and the proportion of time spent resting was highest in summer. Resting decreased and traveling increased in the afternoon.

**Conclusions/Significance:**

Responses to tour vessel traffic are similar to those described for dusky dolphins elsewhere. Disturbance linked to vessels may interrupt social interactions, carry energetic costs, or otherwise affect individual fitness. Research is needed to determine if increased milling is a result of acoustic masking of communication due to vessel noise, and to establish levels at which changes to behavioural budgets of dusky dolphins are likely to cause long-term harm. Threshold values from these studies would allow managers to set appropriate operational conditions based on quantifiable criteria.

## Introduction

Commercial cetacean-watching has grown substantially in the last two decades, resulting in global annual revenues of greater than US$2.1 billion from more than 13 million participants in 2008 [1]. Over 13,000 people are employed by more than 3000 whale-watching operations in 119 countries [1], and research indicates there is still substantial room left for growth in the industry [2].

As the cetacean-watching industry has grown, questions have been raised regarding widespread, high-intensity tourism and potential effects on health and well-being of wild cetacean populations [3]. Questions of reduced fitness, increased sound levels, sensitisation, displacement, and long-term effects of stress were not considered in the early days of the whale-watch industry, since these effects were believed to be miniscule relative to the harm inflicted upon animals by hunting [1]. In the intervening years, substantial interest has been generated within the scientific community to determine effects of tourism on cetaceans and whether the activity is sustainable, particularly as the number and size of vessels have increased [4], [5].

Research has demonstrated a variety of short-term effects on cetaceans in the presence of vessel traffic, including changes in swimming speed and direction [6]–[12], habitat use [13]–[16], communication [17]–[20], distance between individuals [8], [13], [21], [22], respiration and dive characteristics [7], [8], [23]–[25], and activity [3], [14], [26]–[31]. Studies of short-term effects have become an important tool in managing cetacean tourism, though these changes do not offer direct evidence of population-level impacts [32], [33]. Scientists often view such responses to disturbance as a cautionary signal that human activity may be harming the focal species, and studies have linked exposure to tour vessels with long-term effects such as area displacement [14], reduced reproductive success [32], and declining populations [34]. In the absence of studies to link these short-term effects to long-term impacts (or lack thereof), scientists and wildlife managers must generally rely upon the precautionary principle [35] to support decisions to limit or reduce vessel traffic. While this is useful in the absence of better information, management decisions based on quantitative criteria developed as part of an integrated, adaptive management scheme offer more robust protection and are less likely to be affected by short-term economic or political conditions [36].

**Figure 1:**
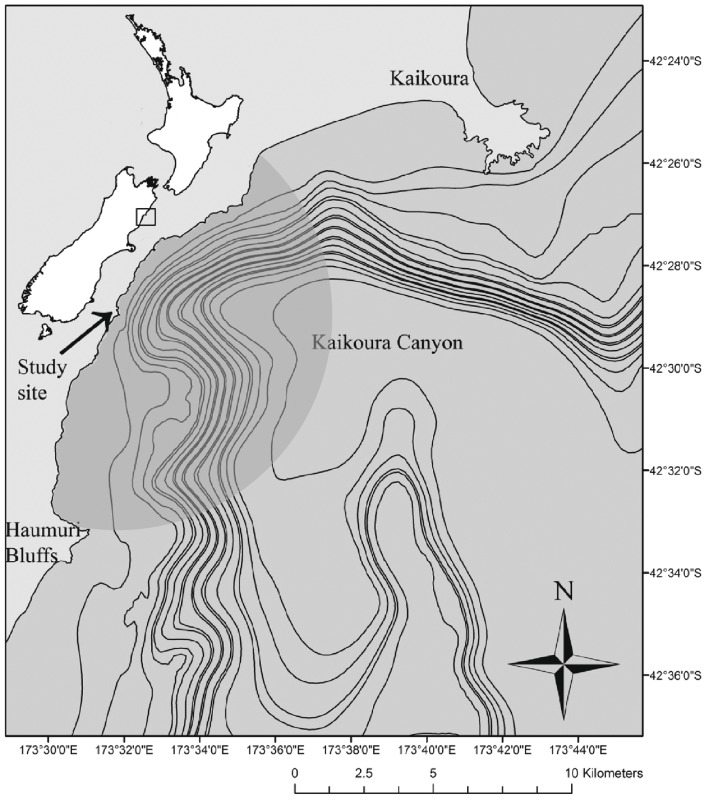
Study area from Kaikoura Peninsula to Haumuri Bluffs. Lines are 100 m depth contours. Location of theodolite station used to collect data indicated with arrow. Gray semi-circle indicates approximate observation area.

Dusky dolphins (*Lagenorhynchus obscurus*) near Kaikoura, New Zealand (Figure 1) are the focus of three forms of tourism. The main source of interactions is from a single company that has permits to use three boats to take tourists to swim with dolphins. Another company has four permits to swim with and watch dolphins and whales, but focuses on viewing sperm whales. These whale-watching boats approach dolphin pods when they may be conveniently visited in the course of a whale-watching tour, and the tourists never enter the water. Three other companies have permits to watch dolphins from the air using fixed-wing airplanes (two companies) and helicopters (one company).

Shore- and boat-based observations have been used for more than 25 years by researchers to track dusky dolphin groups (summarised in [37]) near Kaikoura, beginning with studies of distribution and movement patterns in the mid-1980s prior to the advent of dolphin tourism in the area [38]. Studies of the short-term responses of dusky dolphins to tourist vessels in the mid-1990s found increased vocalisation and activity levels in the presence of swimmers [17], increased aerial activity and decreased inter-individual distance in the presence of vessels [27], [39], and movement away from the direction in which tour boats approach [40]. These studies led to a ten-year moratorium on new commercial marine mammal viewing permits beginning in 1999. The present study was conducted to investigate the effects of current tourism activity on the behavioural budget of dusky dolphins near Kaikoura across seasons and at different times of day. Knowledge gained here can be used to inform current management decision-making and also to facilitate development of quantitative criteria for management of this industry in the future.

## Materials and Methods

Data were collected from January 2008 through January 2010 from an elevated shore station (72.6 m) located just south of Goose Bay, roughly halfway between the Kaikoura Peninsula and the Haumuri Bluffs (Figure 1). The site is located near to the head of the Kaikoura Canyon system, where dolphins often re-group in the morning after nighttime feeding in deep offshore waters [41]. Tracking from shore provided a vantage for determining the behavioural state of dolphin groups within 10 km of the station (in good visibility), while ensuring that the behaviour of dolphins was not affected by the observer.

Focal group follow methods [42] were used to track large pods (>100 individuals) of dusky dolphins from shore, as these are the groups targeted by tour vessels. The research team generally consisted of three people: a primary observer (always DL) to track behaviour and positions of dolphins and vessels, a spotter using a telescope to search for other pods or vessels, and a computer operator to enter the data. Dolphin groups were defined using a 10 m chain rule [43]. Any two dolphins within 10 m of each other were considered part of the same group. This chain was then built to include members spread out over a large area, as long as each individual was within 10 m of another. Choosing the focal group was generally simple, as most often there was a single large group in the study area. When multiple groups were present in the study area, all would be tracked, with the spotter assisting in determining behaviour. The focal follow continued until the focal group could no longer be tracked due to distance, conditions (rain, wind, Beaufort >3), or end of usable daylight.

Data were recorded every 150 s, a longer interval than used by some researchers [17], [40] and shorter than others [39]. This interval was chosen because it was long enough to collect data when multiple vessels were interacting with the focal group (i.e., busy times), and short enough to capture behavioural responses to vessels approaching. Predominant group activity was recorded as the behavioural state (Table 1) for the group [47]. This was accomplished by scan sampling [42] the focal pod to determine the activity state of the majority of the individuals. This method is useful when observing large groups, as the general activity level of the whole group can be described consistently [42]. The number of vessels within 300 m of the group was recorded, as well as the presence of aircraft lower than 1000 ft and within 300 m horizontally from the pod. These are the threshold limits within which vessels are considered to be interacting with dolphins (New Zealand Marine Mammals Protection Regulations 1992, SR 1992/322; http://www.legislation.govt.nz/regulation/public/1992/0322/latest/whole.html).

**Table 1:** Definitions of group behavioral states of dusky dolphins.

State	Definition
Resting	Dolphins close to the surface and each other, surfacing at regular intervals and in a coordinated fashion. Movement very slow.
Traveling	All individuals oriented and moving in the same direction. This behavioural state includes all high-speed, directional behaviours (e.g. porpoising).
Milling	Individuals within the group simultaneously moving in different directions, with no overall clear direction of travel.
Socialising	Physical interactions taking place among members of the group, including chasing, high levels of body contact, coordinated clean leaps and noisy leaps [27].
Feeding	Dolphins observed either capturing or pursuing fish. High number of non-coordinated re-entry leaps, rapid changes in direction and long dives. Seabirds often seen diving among pod.

Adapted from [44]–[46].

### Data analysis

Since consecutive behavioural observations are not likely to be statistically independent, they were analysed as a series of time-discrete Markov chains [48]. First-order Markov chain analyses were used to quantify the dependence of each behaviour event on the preceding event in the behavioural sequence. Following the assumptions used by [49], defining a set of mutually exclusive and wholly inclusive behaviours (Table 1) permits analysis of variation in behaviour of dolphin groups using Markov chains.

Analysis of vessel-caused differences in behaviour was complicated by the dolphins' normal seasonal and diurnal patterns of behaviour [37], [38], [50]. Therefore, each 150 s sample was classified according to the behavioural state, number of vessels (including aircraft) present, and season (based on the date: Dec-Feb  =  Summer, Mar-May  =  Autumn, Jun-Aug  =  Winter, Sept-Nov  =  Spring). To account for varying length of daylight across the year, a time of day index was calculated as the difference between the time of the sample and sunrise divided by the length of daylight (time of sunset – time of sunrise). This index represented a percentile of daylight hours where sunrise  = 0, midday  = 0.5, and sunset  = 1.0. This was used to classify each sample as morning (<0.33), midday (0.33 to 0.66), or afternoon (>0.66).

Markov chains were used to build transition matrices of preceding behaviour (at time 0) versus succeeding behaviour (at time 1) for each transition within the Control (no vessels present) and Treatment (while vessels were present within 300 m of the focal dolphin group) chains, split by season and time of day. A transition was only included in the Control chain when no vessels were present for at least 15 min prior to the observation period in order to reduce the likelihood that dolphin behaviour was altered due to a vessel interaction. Sample size limitations made it impossible to build matrices with different numbers of vessels, so a simple presence/absence analysis was performed to determine whether vessels had a significant effect on behavioural transitions of dolphins.

Ideally, a Before-After-Control-Impact (BACI) approach would be used to analyse dolphin responses to vessel traffic [51]. This approach requires monitoring responses at both control (no traffic) and impact (traffic) sites. Unfortunately, the Kaikoura Canyon system has unique geography which leads to different behavioural ecology of dusky dolphins in Kaikoura than in other areas of New Zealand [52], so no similar site exists to use as a control. The next most appropriate research design is a Before-During-After (BDA) approach, where responses are compared at a single site, prior to the vessel approach, during the interaction, and after the vessel departs [51]. However, due to the frequency of vessel and aircraft approaches to dusky dolphins in Kaikoura, a BDA analytical approach was difficult because dolphins were rarely unaccompanied for sufficient time for any potential behavioural change to subside [27], [50], [53]. Especially in summer, when early morning dolphin-swim tours were on the water before sunrise, it often was not possible to locate and observe dolphins before vessels approached the group.

Log-linear analysis (LLA) was conducted in *R* [54] to test whether the presence of vessels altered the likelihood of dolphins moving from one behavioural state to another. This was accomplished by using count data from the transition matrices and testing models in *R* for all combinations of parameters and interactions between parameters: 5 preceding behaviours ×5 succeeding behaviours ×4 seasons ×2 vessel conditions. Maximum likelihood for the model being tested was approximated by *G^2^*. Comparing the goodness-of-fit for each model to the goodness-of-fit for the fully saturated model (*?G^2^*) approximated the effect of the missing variables [49]. Degrees of freedom were the difference in degrees of freedom between the two models. Evaluating the significance of this difference determined which variables were significant. Akaike Information Criteria (AIC) [55] values were calculated and used to choose the best-fitting model.

Based on the results from the LLA, the transition matrices were used to calculate the behavioural budget of dusky dolphins in the absence and presence of vessels by season and time of day. Following [49], the left eigenvector of the dominant eigenvalue of each transition matrix was used to approximate the behavioural budget of dusky dolphins under the conditions of the matrix. A Z test for proportions [56] was used to test for differences between behavioural budgets, and 95% confidence intervals (CI) were calculated.

## Results

Dolphins were successfully tracked from shore on 220 d from January 2008 through January 2010. A total of 728 h of tracks were collected for 404 dolphin groups. Spotting effort was equal across all seasons, but more dolphin groups were found in summer (231 h, 53 d, 148 groups) and autumn (246 h, 53 d, 126 groups) than in spring (158 h, 51 d, 78 groups) or winter (93 h, 35 d, 52 groups). A total of 1203 vessels were observed within 300 m of dolphins, with uneven distribution across season (summer  = 403, autumn  = 422, spring  = 253, and winter  = 125). Vessels were within 300 m of dolphins 55% of the time in summer (mean ± SE # vessels when present  = 1.99±0.02), 52% of the time in autumn (1.87±0.02), 49% of the time in spring (1.90±0.02), and 44% of the time in winter (1.21±0.02). Vessels were present 51% of the time in the first third of the day (1.84±0.02), 53% of the time in the second third (1.90±0.02), and 49% of the time in the final third (1.73±0.03).

**Figure 2:**
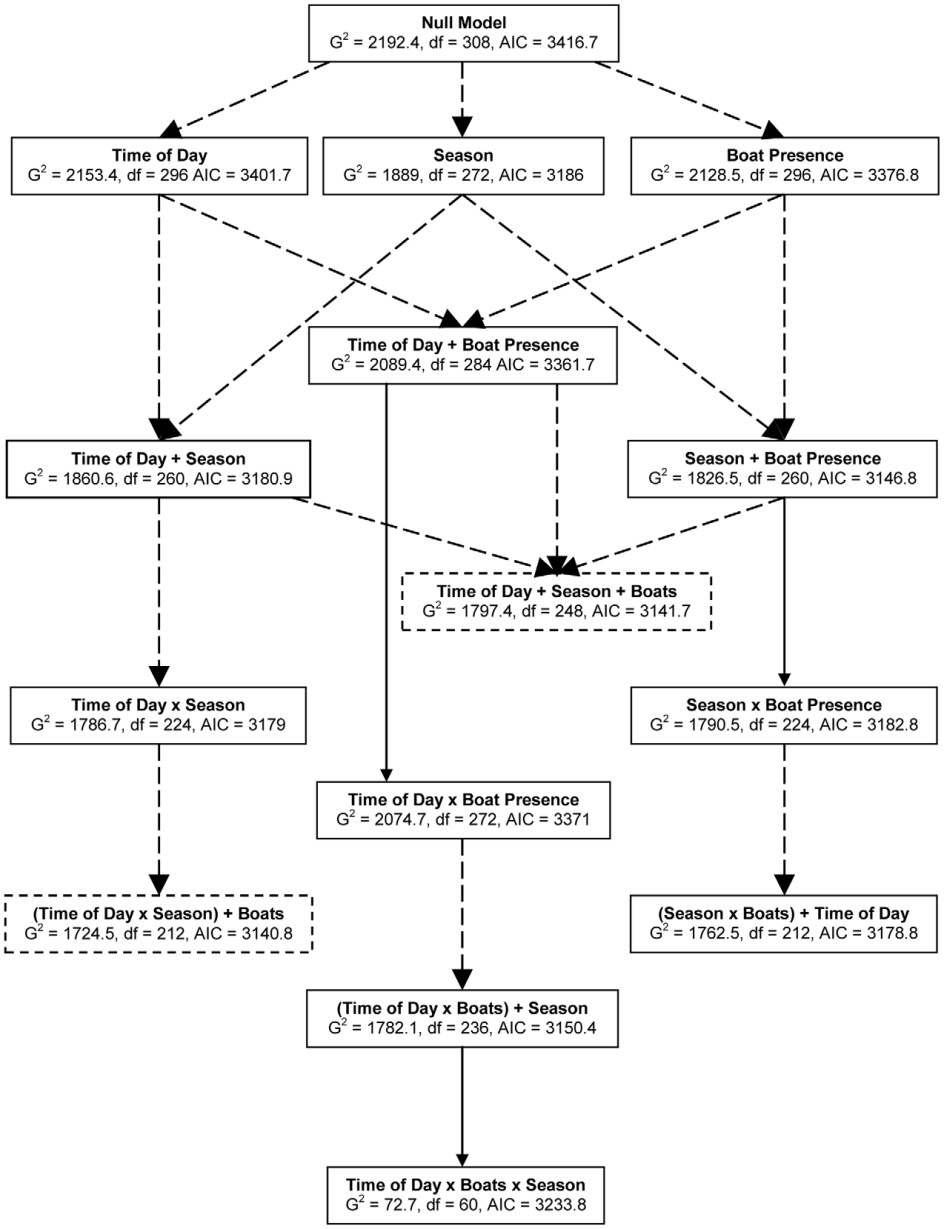
Effects of vessel presence, time of day, and seasons on behavioural state transitions. The null model (no effects due to the three factors) is at the top, and each branch below adds an effect due to a factor or interaction between factors. Boxes represent the model tested, with *G^2^*, degrees of freedom (df), and Akaike Information Criteria (AIC) values listed. An ‘x’ between two terms indicates that their interaction is included. Dashed arrows indicate significant terms added (p<0.05). Dashed boxes indicate the best fitting models determined by AIC values.

A total of 11,373 behavioural transitions were observed. The breakdown of behavioural state transitions by season, time of day, and vessel presence is shown in Table 2. Dusky dolphins at Kaikoura typically feed at night [41], and therefore group behavioural state was seldom recorded as feeding (<0.5% of observations). Therefore, this behavioural state was removed from the analysis. The remaining counts were sufficient to conduct a full 5-way LLA of the effects of these factors on behavioural transitions. Results of this analysis are shown graphically in Figure 2. Significant differences are found when each of the factors is added to the null model (Figure 2), indicating that dolphin behaviour changes due to natural (time of day, season) and anthropogenic (vessel presence) factors. Comparison of AIC values reveals that two models provide more information than the others (Figure 2). Both include effects due to vessels, time of day, and season, and the one with the greatest likelihood also includes the interaction between time of day and season (Figure 2).

**Table 2:** Count of observed behavioural state transitions by season, time of day, and vessel presence.

	Morning		Midday		Afternoon	
	Vessel	No Vessel	Vessel	No Vessel	Vessel	No Vessel
Autumn	392	433	625	417	241	324
Spring	528	677	777	593	102	168
Summer	959	644	1090	1111	447	219
Winter	158	184	325	368	230	361

Morning, midday, and afternoon each account for one-third of the hours from sunrise to sunset.

**Figure 3:**
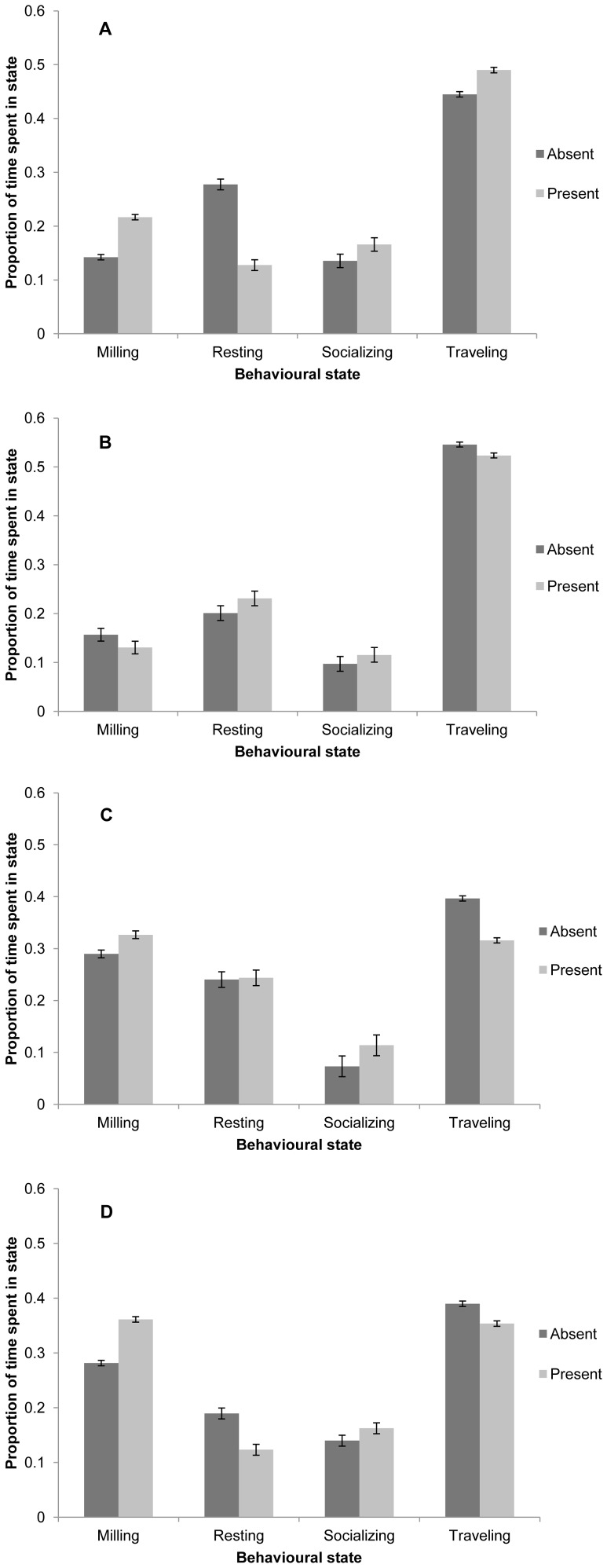
Seasonal behavioural budget of dusky dolphin groups in the presence and absence of vessels. Behavioural budget split by A) Summer, B) Autumn, C) Winter, and D) Spring. Error bars are 95% confidence intervals.

Markov-chain analysis revealed that the behavioural budget of dusky dolphins was affected by the presence of vessels differently depending on season (Figure 3). The proportion of time spent milling significantly increased in the presence of vessels in all seasons except autumn (Figure 3). Resting significantly declined when vessels were present in spring and summer (Figure 3). The proportion of time spent traveling significantly decreased in the presence of vessels in all seasons except summer, when it increased (Figure 3).

**Figure 4:**
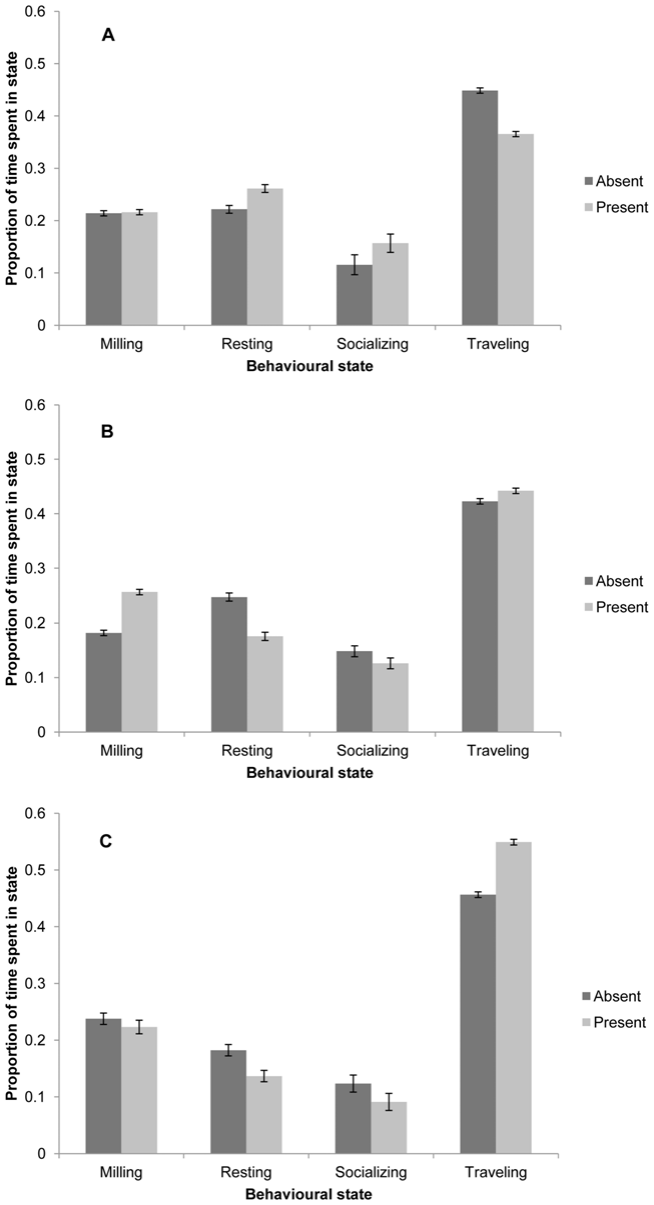
Diurnal variation in the behavioural budget of dolphin groups in the presence and absence of vessels. Behavioural budget split by A) Morning, B) Midday, and C) Afternoon. Error bars are 95% confidence intervals.

Dusky dolphins also responded differently to vessel presence depending on time of day (Figure 4). In the morning, dusky dolphins spent a greater proportion of time resting when vessels were present, and less time traveling (Figure 4a). At midday, the proportion of time spent resting significantly decreased in the presence of vessels, while significantly more time was spent milling and traveling (Figure 4b). In the afternoon, the proportion of time spent resting significantly decreased in the presence of vessels, and significantly more time was spent traveling (Figure 4c).

## Discussion

Seasonal and diurnal variation in dusky dolphin behaviour outlined here correlates well with that described by previous researchers. Calves are born and conception generally occurs in late spring and early summer [38], when socialising is more frequently seen. Summer is characterized by elevated levels of rest, coinciding with the presence of young calves in the area [38]. Individual dolphins are seasonally resident in this area [50], and elevated levels of traveling in autumn may be associated with movement of groups to and from the area. Dolphins move offshore in the late afternoon [38], [50] in preparation to feed at night in deep water over the Kaikoura Canyon system [41], which is reflected by an increase in traveling. Dolphins which have spread out and separated over a night of foraging must re-form groups in the early morning as individuals move back to shallow, near-shore waters. A period of low activity then occurs from late morning to midday [38], [39], indicated by elevated levels of rest.

Using powerful maximum likelihood-based analysis techniques, it was possible to describe how the activity budget of dolphins changes in the presence of vessels seasonally and at different times of day. Resting significantly decreased in the presence of vessels in spring and summer, when calves are most likely to be present [38]. A particularly large reduction in resting in the presence of vessels was seen in summer, the season where resting is most common. It is possible that this reduction is due to the greater number of vessels interacting with dolphins in busy summer months [50], [53], which may also explain why summer is the only season where significantly higher levels of traveling are seen in the presence of vessels. Dolphins spent more time resting and less time traveling in the presence of vessels in the morning, but at midday and in the afternoon the exact opposite was found. This shift in response to interactions during the course of the day may also be explained by diurnal patterns of dolphin behaviour. Dolphins which have fed at night [41] and are re-forming social groups early in the morning react differently to vessel interactions than those that are resting at midday [38], [39] or moving offshore in the afternoon.

### Potential consequences of short-term changes

#### Energetic costs

It has been suggested that short-term changes in behaviour could result in negative energetic effects on cetacean populations that are the focus of tourism operations [57]. If time is spent interacting with vessels (or avoiding them) rather than foraging, socialising, or resting, individual fitness may be reduced, leading to population-level effects [32]. A study of dusky dolphin responses to tourism in Patagonia, Argentina found that feeding and socialising decreased while milling increased in the presence of tour vessels [29]. The dolphin groups that were tracked in Patagonia were smaller than those studied in the present project. The main difference is that feeding was reduced in Argentina whereas resting was reduced in New Zealand. These two activities, however, both have the potential to affect the energetic balance of dolphins, if disturbance is chronic and of high intensity.

Dusky dolphins in the Kaikoura area primarily feed offshore on a deep scattering layer (DSL) of mesopelagic fishes and squid [41]. The DSL only rises near enough to the surface for efficient feeding at night, when no vessels are directly targeting dolphins. Opportunistic feeding occurs during the day, but is rare in comparison to foraging rates described in Patagonia [29], or even dusky dolphins in other parts of New Zealand [50]. Foraging activity in Kaikoura may therefore be protected by the difference between the timing and location of feeding versus the timing and location of tourism activity. Increased milling in the presence of vessels is an ambiguous result relative to the energetic balance of individuals. For instance, overall speed of travel for the group may decrease at the same time that individual speeds (and thus individual energy expenditure) increase, if dolphins are swimming rapidly in different directions.

Dusky dolphins are highly mobile, moving between areas of New Zealand at different times of year [50], and exhibit a wide variation in behaviour by season and time of day. Thus, an increase in energy expenditure due to tourism vessels might be small in relation to normal energy budget variation, and not be expected to result in population-level effects. However, if this increase is combined with a simultaneous reduction in resting, there is more reason for concern. Rest is one of the primary daytime behavioural states at Kaikoura (∼25% of daylight hours), and during this study was partially protected by a midday rest period wherein commercial operators voluntarily refrained from approaching dolphin groups between 11∶30 am and 1∶30 pm from December through March. Compliance varied by operator [58], and in 2010 new regulations were instituted which made the rest period mandatory from November through February and voluntary in March (A. Baxter, New Zealand Department of Conservation, personal communication). However, non-commercial vessels may still approach dolphins during this time, and dolphins at rest may be disturbed by vessel traffic outside these hours, so more information is needed to determine whether this rest period provides appropriate protection from the energetic costs of disturbance. A study is needed to elucidate energy consumption of dusky dolphins in different behavioural states. These data could be combined with the activity budget differences reported here to determine the energetic cost of vessel presence. These results could then be used in conjunction with estimates of the overall daily energetic budget of dusky dolphins [38], [59] to determine threshold values at which negative impacts might be expected, and management guidelines set based on these thresholds.

#### Acoustic disturbance

Research suggests that when animal groups are moving, a few individuals within the group are able to direct the movement of the whole group [60]. Noise from vessel motors may mask communication [61] and lead to confusion within the group, as the individuals with knowledge (where food is located, where predators are located, which areas offer protection) cannot effectively communicate with other members of the group. Therefore, acoustic disturbance has the potential to expose individuals to increased risk of predation by large sharks and killer whales [62], reduce foraging efficiency [29], and lead to reduced individual fitness. It is possible that acoustic masking is occurring with dusky dolphins at Kaikoura, as evidenced by increased levels of milling, a sign the group may no longer be able to efficiently reach a consensus on direction of movement. A study which investigates vocal behaviour of dusky dolphins in the absence and presence of vessel traffic would be very valuable in determining whether acoustic masking occurs, and at what levels of sound. If masking occurs, appropriate management limits on source sound levels and characteristics could be set.

### Conclusions and future work

Dusky dolphins off Kaikoura occur in large numbers [37], each individual is only present in Kaikoura part of the year [50], the dolphins feed at night when vessels are not present [41], and individuals have the ability to move away from tourism areas from day to day. All of these factors likely reduce the effects incurred by each individual dolphin and would be expected to lend some resilience to the effects of vessel traffic. Management restricting the number of commercial tours and vessels may help minimise effects. Mandatory regulations are the best way to minimise potentially negative effects. As a result of recommendations made by researchers [63], a number of operating changes were made by the Department of Conservation in an attempt to reduce the number and duration of vessel interactions with dusky dolphins. The maximum number of swimmers permitted per vessel was raised from 13 to 16 (A. Baxter, personal communication), thus encouraging operators to use 2 vessels rather than three when total swimmer numbers are between 27 and 32. The number of times swimmers are allowed to enter the water was limited to five per vessel per trip (A. Baxter, personal communication), to limit the intensity and duration of any single tour, particularly when dolphins are uninterested or avoiding interactions. Additionally, a 5-year moratorium on additional marine mammal viewing permits was instituted (A. Baxter, personal communication).

While short-term changes in the behaviour of dolphins are evident, it is not immediately obvious that these effects are detrimental in the long-term. Population-level effects are difficult to detect for marine mammals, and particularly so for a large and mobile population [50] such as dusky dolphins in New Zealand. The only photographic mark-recapture estimate of this population [50] resulted in a population size estimate of 12,626 animals, not including calves. No standard error was provided with this number, but given the high percentage of unmarked animals (38%) [50], it is reasonable to assume that the actual population may be plus or minus several thousand individuals. Similar estimates are only likely to detect population-level changes if effects are very large, but disturbance due to tourism is not likely to result in population changes of this magnitude. Therefore, management of this activity has focused on observing short-term behavioural responses and applying the precautionary principle to reduce the potential negative effects of these changes. In order to move forward and build a management scheme with robust, quantifiable criteria, research is needed into the likely mechanisms of disturbance (such as vessel noise) and physiological effects on the target species (such as altered energetic balance and levels of stress hormones).

An energetic study could be accomplished in multiple ways. The field metabolic rate of dusky dolphins in different behavioural states could be estimated by injecting animals with doubly labelled water (DLW) and taking blood samples (see [64] for a description of this technique), though this may or may not be a viable option with marine mammals [65]. Attaching or implanting a tag which records heart rate can be used to estimate metabolic rates [65] and has been used in marine mammals [66], though creating an appropriate calibration curve would most likely require captive animals. Alternatively, a suction cup tag that records the number and relative strength of fluke strokes could be used as a less explicit method of estimating the relative energy consumption in different behavioural states.

Acoustic disturbance of dusky dolphins could be studied by attaching a tag that records vocalisations of the individual to which it is attached, conspecifics, and vessel noise. One study attached data tags to northern elephant seals [67]; the tags collected dive pattern, ambient and vessel noise, respiration, heart rate, and vocalisation data. Using such a tag with controlled boat interactions would allow analysis of a number of effects, including whether vocalising changes in the presence of a vessel, the received sound level at which changes occur, and whether this changes when multiple vessels are present. Fecal hormone levels may be used to assess reproductive status and function in cetaceans [68]. Elevated levels of glucocorticoid hormone metabolites were found in the faeces of northern right whales (*Eubalaena glacialis*) exposed to noise from shipping traffic [69]. This non-invasive sampling method allows quantification of mean levels of stress hormones, and could be used to study the effect of tourism on dolphins by comparing hormone levels between tourism areas and non-tourism areas. Studies such as these would provide wildlife managers with valuable information for setting appropriate operational conditions and quantifiable criteria for evaluating changes over time.
